# Estimation of lung tumor position from multiple anatomical features on 4D‐CT using multiple regression analysis

**DOI:** 10.1002/acm2.12121

**Published:** 2017-06-29

**Authors:** Tomohiro Ono, Mitsuhiro Nakamura, Yoshinori Hirose, Kenji Kitsuda, Yuka Ono, Takashi Ishigaki, Masahiro Hiraoka

**Affiliations:** ^1^ Department of Radiation Oncology and Image‐applied Therapy Graduate School of Medicine Kyoto University Kyoto Japan; ^2^ Division of Radiology Osaka Red Cross Hospital Osaka Japan; ^3^ Department of Radiation Oncology Osaka Red Cross Hospital Osaka Japan

**Keywords:** 4D‐CT, anatomical features, estimation of lung tumor position, multi regression analysis

## Abstract

To estimate the lung tumor position from multiple anatomical features on four‐dimensional computed tomography (4D‐CT) data sets using single regression analysis (SRA) and multiple regression analysis (MRA) approach and evaluate an impact of the approach on internal target volume (ITV) for stereotactic body radiotherapy (SBRT) of the lung. Eleven consecutive lung cancer patients (12 cases) underwent 4D‐CT scanning. The three‐dimensional (3D) lung tumor motion exceeded 5 mm. The 3D tumor position and anatomical features, including lung volume, diaphragm, abdominal wall, and chest wall positions, were measured on 4D‐CT images. The tumor position was estimated by SRA using each anatomical feature and MRA using all anatomical features. The difference between the actual and estimated tumor positions was defined as the root‐mean‐square error (RMSE). A standard partial regression coefficient for the MRA was evaluated. The 3D lung tumor position showed a high correlation with the lung volume (R = 0.92 ± 0.10). Additionally, ITVs derived from SRA and MRA approaches were compared with ITV derived from contouring gross tumor volumes on all 10 phases of the 4D‐CT (conventional ITV). The RMSE of the SRA was within 3.7 mm in all directions. Also, the RMSE of the MRA was within 1.6 mm in all directions. The standard partial regression coefficient for the lung volume was the largest and had the most influence on the estimated tumor position. Compared with conventional ITV, average percentage decrease of ITV were 31.9% and 38.3% using SRA and MRA approaches, respectively. The estimation accuracy of lung tumor position was improved by the MRA approach, which provided smaller ITV than conventional ITV.

## 1 INTRODUCTION

Lung cancer is the leading cause of cancer‐related death worldwide^1^ The standard treatment for patients with lung cancer is surgical resection; however, radiotherapy has come to play an increasingly important role for patients who are unable to undergo any type of surgery, especially those with early‐stage lung cancer.^2^

When treating lung cancer with radiotherapy, respiratory motion is one of the factors causing uncertainties during treatment planning and beam delivery. Without respiratory motion management, larger margins would be needed to account for respiratory motion, leading to a larger planning target volume (PTV) size, which, in turn, includes a larger organ at risk volume. Matsuo et al. examined the relationship between frequency of normal tissue toxicity and PTV size^3^ showing that the frequency of symptomatic radiation pneumonitis was significantly lower with a PTV size of <37.7 mL than with a PTV size of ≥37.7 mL (11.1% vs. 34.5%) for nonsmall cell lung cancer with stereotactic body radiotherapy (SBRT). Thus, to reduce the dose to the normal lung is of clinical importance in terms of toxicity.

The American Association of Physicists in Medicine Task Group 76 has advocated an emphasis on respiratory motion management^4^ Four‐dimensional computed tomography (4D‐CT) and 4D radiotherapy (4D‐RT) provide patient‐specific radiation treatment, taking respiratory‐induced anatomical motion into account. Both approaches frequently require internal and/or external respiratory motion signals. Generally, image quality of 4D‐CT and the advisability of 4D‐RT are dependent on correlations with respiratory motion signals (surrogate signals)^5^, ^6^ That is, whether the surrogate signals represent the target well is very important in 4D‐CT and 4D‐RT.

A high correlation between tumor motion and a surrogate signal such as ventilation volume and abdominal displacement has been reported from several facilities^7^, ^8^ Gianoli et al. used one of multiple infrared markers placed on the thoracoabdominal surface to obtain high‐quality 4D‐CT images and showed that the 4D‐CT image quality was improved, using a multidimensional K‐means clustering method.^9^ Thus, one surrogate signal acquired from one anatomical feature is frequently used in 4D‐CT and 4D‐RT. However, no correlation between tumor motion and a surrogate signal for one anatomical feature would cause a decrease in 4D‐CT image quality or erroneous irradiation. Therefore, the use of multiple surrogate signals for several anatomical features could reduce such risks.

In the current study, we estimated lung tumor position from multiple anatomical features acquired from 4D‐CT image sets, including the lung volume, the displacement of diaphragm position, abdominal wall position, and chest wall position, using multiple regression analysis (MRA) and single regression analysis (SRA) approaches. In addition, we assessed an impact of these approaches on internal target volume (ITV) for SBRT of the lung.

## 2 METHODS

### 2.A Patients and data acquisition

Of the patients who underwent SBRT at Osaka Red Cross Hospital between November 2011 and April 2015, 11 consecutive lung cancer patients (12 cases) with three‐dimensional (3D) motion ranges greater than 5 mm were enrolled in this study. There were seven men and four women with a median age of 76 (range, 67–98) yr. Lung tumors were located in the right upper lobe (two patients), in the right middle lobe (two patients), in the right lower lobe (six patients), and in the left lower lobe (two patients). 4D‐CT data were acquired using the Discovery CT750HD (GE Medical Systems, Waukesha, WI, USA) and the Real‐time Positioning Management (RPM) system (Varian Medical Systems, Palo Alto, CA, USA) in axial cine mode for all patients. 4D‐CT was performed under free breathing without audio/visual coaching. The CT slice thickness was 2.5 mm. Cine duration time of the scan at each couch position was set to 0.5 s, which was more than the maximum observed respiratory period. The cine interval between images was 1.3 s. CT data were reconstructed in a field of view of 500 mm on a 512 × 512 grid for the 4D‐CT scan. The RPM system illuminated and tracked an infrared reflective marker placed on the patient's abdomen. The RPM software was used to calculate the respiratory phase at each instant in time based on modeling the abdominal motion amplitude. The RPM system was used to calculate a phase at each point of a respiratory trace, where 0% corresponded to the inhalation peak and 50% to the midpoint between consecutive inhalation peaks. All CT slices and the RPM respiratory data file were transferred to an Advantage 4D workstation (GE). The Advantage 4D software was used to read all CT slices as well as the corresponding RPM respiratory data file, to assign a phase to each CT slice, according to the temporal correlation between the RPM trace and CT data acquisition, and to export 10 respiratory phase volumes, evenly distributed over a respiratory cycle.

### 2.B Lung tumor position and anatomical features

Lung tumors were delineated manually by two radiation oncologists for all phases. Lung tumor positions, defined by the center of volume, were recorded in left‐right (LR), anterior‐posterior (AP), and superior‐inferior (SI) directions. As anatomical features, lung volume and displacement of the diaphragm, abdominal wall, and chest wall were extracted from all phases. Lung volume was extracted using auto‐thresholding, and displacement of the diaphragm position in the SI direction and of the abdominal and chest wall positions in the AP direction was determined on the treatment planning system (Eclipse; Varian). Displacement of the diaphragm, abdominal wall, and chest wall were measured at the top of the diaphragm in the SI direction, at the level of the umbilicus in the AP direction, and at the level of the junctional region between the sternum and the xiphisternum in the AP direction, respectively.

Then, Pearson's correlation coefficient (CC) was calculated between the lung tumor position and the four anatomical features: lung volume and displacements of the diaphragm, abdominal wall, and chest wall for each phase in each patient. In addition, the coefficient of variation (CV) was used to evaluate variation in the gross tumor volume (GTV) because of motion artifacts and tumor deformation during respiration.

### 2.C Single or multiple regression analysis

Lung tumor positions were estimated from anatomical features using SRA and MRA approaches. SRA is a statistical approach to estimate one objective variable from one explanatory variable. The MRA approach estimates one objective variable from multiple explanatory variables. In this study, the lung tumor position was defined as the objective variable, and lung volume and displacements of the diaphragm, abdominal wall, and chest wall were defined as the explanatory variables. The estimated lung tumor position (X_E_, Y_E_, Z_E_) was calculated from the following equation:

(1) $$\left(\begin{array}{c} X_{E}\\ Y_{E}\\ Z_{E} \end{array}\right)=\left(\begin{array}{c} a_{L_{1}}x_{L}+b_{D_{1}}x_{D}+c_{A_{1}}x_{A}+d_{C_{1}}x_{C}+e_{1}\\ a_{L_{2}}x_{L}+b_{D_{2}}x_{D}+c_{A_{2}}x_{A}+d_{C_{2}}x_{C}+e_{2}\\ a_{L_{3}}x_{L}+b_{D_{3}}x_{D}+c_{A_{3}}x_{A}+d_{C_{3}}x_{C}+e_{3} \end{array}\right)$$

where x_L_, x_D_, x_A_, and x_C_ are the explanatory variables of lung volume and displacements of the diaphragm, abdominal wall, and chest wall, respectively. The estimated lung tumor position in the X_E_, Y_E_, and Z_E_ directions indicates the LR, AP, and SI directions, respectively. The coefficients of *a* to *d* are partial regression coefficients and independent of each direction. Also, *e*_*1*_ *, e*_*2*_ *and e*_*3*_ were a constant term. When performing the SRA approach, lung tumor position was estimated using one explanatory variable. For example, when the lung tumor position was estimated from the one explanatory variable of lung volume, a single coefficient of *a* was used, and other coefficients of *b* to *d* were zero. Consequently, four results of SRA and one MRA were analyzed for all cases.

To evaluate estimation approaches for SRA and MRA, the following three analyses were performed. First, a coefficient of determination was evaluated to explain how well an explanatory variable fitted an objective variable. Using a value of 0 to 1, the coefficient of determination was calculated as the square of the CC between the lung tumor position and anatomical features. Adjusted coefficient of determination was used. Second, the difference between actual and estimated lung tumor position was defined as a root‐mean‐square error (RMSE). After measuring the lung tumor position, the difference was evaluated in an absolute manner and a relative manner. The relative manner was expressed as a percentage of the displacement of the actual lung tumor position. Finally, the standard partial regression coefficient was calculated to evaluate the influence of each explanatory variable on the estimated lung tumor position. The standard partial regression coefficient is the standardized value of the partial regression coefficient and denotes the relative importance of an explanatory variable. The largest magnitude of the standard partial regression coefficient has the most influence on the objective value. The standard partial regression coefficient was calculated using the following equation:

(2) $$\begin{array}{cc} \text{Standard partial regression coefficient} & =\text{Partial regression coefficient}\\ & \;\times \frac{SD_{obj}}{SD_{exp}} \end{array}$$

where SD_obj_ is the standard deviation (SD) of the objective variable, and SD_exp_ is the standard deviation of the explanatory variable. The standard partial regression coefficient refers to *a* to *d* obtained from eq. (1). In this study, the standard partial regression coefficient was evaluated for four explanatory variables, which were the lung volume, the displacement of diaphragm, abdominal wall, and chest wall. Additionally, Student's *t*‐test was performed to explore the impact of the standard partial regression coefficient for each anatomical feature. A difference was considered statistically significant at the *P* < 0.05 level.

### 2.D Comparison of internal target volumes

ITVs derived from SRA (ITV_SRA_) and MRA (ITV_MRA_) approaches were compared with gold‐marker‐based ITV (ITV_GM_) and ITV derived from contouring GTVs on all 10 phases of the 4D‐CT (ITV_conv_) for real‐time tumor tracking (RTTT) SBRT of the lung. The definitions of ITV_SRA_, ITV_MRA_, and ITV_GM_ generated were as follows;

1. ITV_SRA_: The internal margins derived from RMSE of SRA for the lung volume, the displacement of diaphragm, abdominal wall, and chest wall were added to the GTV delineated on 50% phase of 4D‐CT images. Fifty percent phase was corresponding to the midpoint between consecutive inhalation peaks.
2. ITV_MRA_: The internal margins derived from RMSE of MRA were added to the GTV delineated on 50% phase of 4D‐CT images.
3. ITV_GM_: Based on intrafractional variation between the centroid of tumor and the centroid of fiducial markers reported by Ueki et al.,^10^ the internal margins of 0.6 mm, 0.9 mm, and 0.2 mm in LR, AP, and SI directions, respectively, were added to the GTV delineated on 50% phase of 4D‐CT images.

A total of seven ITVs, including four ITV_SRA_s, one ITV_MRA_, one ITV_GM_, and one ITV_conv_, were generated for each patient. Student's *t*‐test was performed and a difference was considered statistically significant at the *P* < 0.05 level.

## 3 RESULTS

### 3.A Correlation between lung tumor position and anatomical features

The median values of the average displacement of lung tumor positions were 1.7 mm (range, 0.5–3.4 mm), 3.6 mm (range, 1.8–5.7 mm), 9.5 mm (range, 5.1–16.9 mm), and 10.5 mm (range, 7.0–17.4 mm) in the LR, AP, SI, and 3D directions, respectively. The median values of the anatomical features were 378.2 mL (range, 234.0–494.7 mL), 12.3 mm (range, 9.0–15.2 mm), 4.9 mm (range, 2.0–7.5 mm), and 1.0 mm (range, 0.3–2.0 mm) for the lung volume and displacement of the diaphragm, abdominal wall, and chest wall, respectively.

Table 1 shows the CC between lung tumor position and anatomical features. The CC is shown as the mean ± SD for all cases. Anatomical features had strong correlations with lung tumor position in the SI and 3D directions, but no correlation in the LR or AP directions. Among anatomical features, the lung volume had a strong correlation with lung tumor position in the SI and 3D directions. The median value of the GTV was 3.7 mL (range, 1.0–24.5 mL), and the median value of the CV in GTV sizes was 7.7% (range, 5.8–16.6%) for all cases.

**Table 1:** Pearson's correlation coefficient (CC) between lung tumor position and anatomical features. Data are shown as means ± standard deviations

	CC			
	LR	AP	SI	3D
Lung	−0.14 ± 0.64	−0.24 ± 0.55	−0.91 ± 0.09	−0.92 ± 0.10
Diaphragm	0.14 ± 0.61	0.18 ± 0.52	0.87 ± 0.15	0.85 ± 0.16
Abdominal wall	−0.02 ± 0.57	−0.30 ± 0.47	−0.77 ± 0.30	−0.78 ± 0.29
Chest wall	−0.18 ± 0.55	−0.06 ± 0.45	−0.55 ± 0.36	−0.57 ± 0.35

LR, left‐right direction; AP, anterior‐posterior direction; SI, superior‐inferior direction; 3D, three dimensions.

### 3.B Difference between actual and estimated lung tumor position

**Figure 1:**
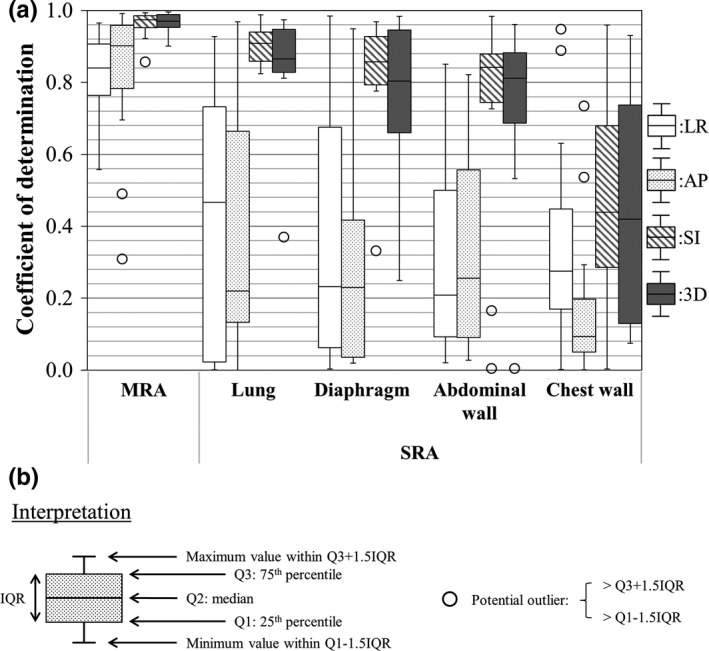
Variations in the coefficient of determination between the lung tumor position and anatomical features for the LR, AP, SI, and 3D directions (a). Interpretations of the boxplots are shown in (b).

**Figure 2:**
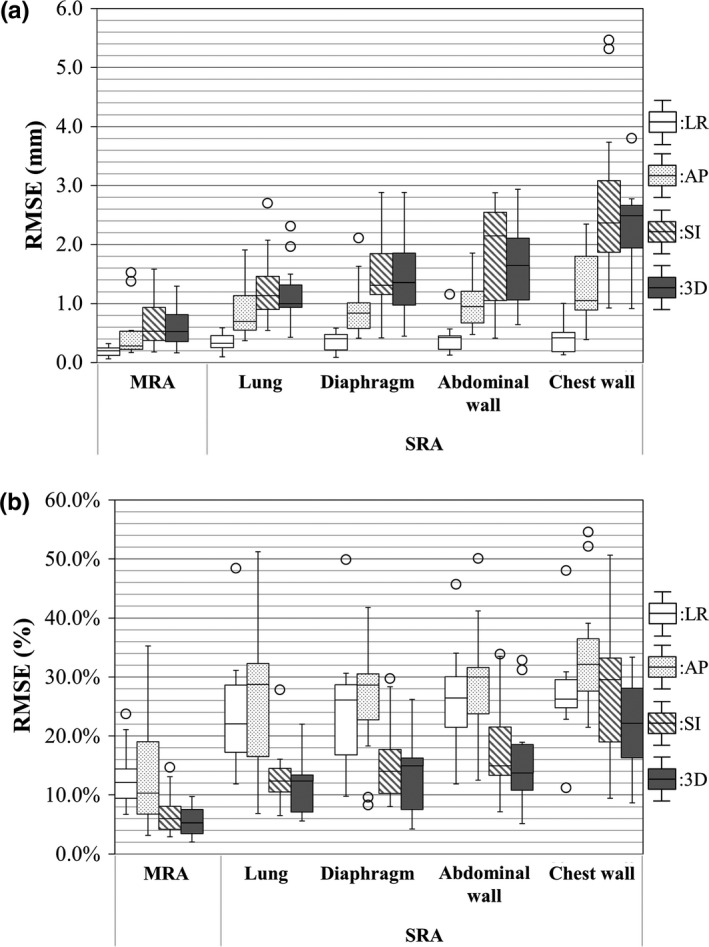
Variations in RMSE between actual and estimated lung tumor positions for the LR, AP, SI, and 3D directions. (a) Absolute manner and (b) relative manner.

**Figure 3:**
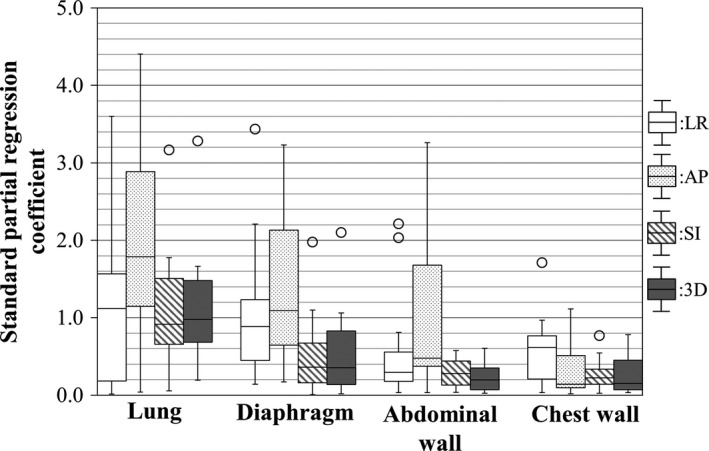
Variations in the standard partial regression coefficient for the LR, AP, SI, and 3D directions.

Figure 1(a) shows the variations in the coefficient of determination between the lung tumor position and anatomical features using the SRA and MRA approaches. Potential outliers were defined as data points falling 1.5‐fold the interquartile range above the upper quartile or below the lower quartile in Fig. 1(b). The MRA approach had a higher correlation with the actual lung tumor position in all directions compared with the SRA approach. Figure 2 shows variations in the RMSE between actual and estimated lung tumor positions using the SRA and MRA approaches as an absolute manner in Fig. 2(a) and a relative manner in Fig. 2(b). In Fig. 2(a), the RMSE of the SRA was within 3.7 mm excluding potential outliers in all directions. Moreover, the RMSE of the MRA was within 1.6 mm in all directions. In both Figs. 2(a) and 2(b), the SRA approach with the lung volume showed the smallest median RMSE. The median RMSE of the chest wall had the largest error in all directions. The MRA approach showed a smaller median RMSE than did any SRA approach in any direction. Figure 3 shows variations in the standard partial regression coefficients using the MRA approach. The standard partial regression coefficient for the lung volume was the largest in all directions. Table 2 shows *P* values of the standard partial regression coefficient. A significant difference indicates that the corresponding anatomical feature had a large influence on estimated lung tumor motion compared with the other anatomical features. Coefficients for the lung volume had significant differences in the 3D direction, compared with other anatomical features.

**Table 2:** *P* values of the standard partial regression coefficient for each anatomical feature

		*P* value			
		LR	AP	SI	3D
Lung	Diaphragm	0.71	0.21	0.06	<0.05
Lung	Abdominal wall	0.14	<0.05	<0.05	<0.05
Lung	Chest wall	0.11	<0.05	<0.05	<0.05
Diaphragm	Abdominal wall	0.19	0.33	0.20	0.08
Diaphragm	Chest wall	0.14	<0.05	0.15	0.13
Abdominal wall	Chest wall	1.00	<0.05	0.75	0.64

LR, left‐right direction; AP, anterior‐posterior direction; SI, superior‐inferior direction; 3D, three dimensions.

### 3.C Comparison of internal target volumes

Table 3 shows sizes of ITV_conv_, ITV_GM_, ITV_SRA_, and ITV_MRA_. Average percentage decrease of ITV compared with ITV_conv_ were 21.3%, 31.9%, 31.9%, 29.8%, 25.5%, and 38.3% for ITV_GM_, ITV_SRA_ derived from the lung volume, the displacement of diaphragm, abdominal wall, and chest wall, and ITV_MRA_, respectively. ITV_GM_, ITV_SRA_, and ITV_MRA_ were significantly smaller than ITV_conv_ (*P* < 0.05).

**Table 3:** Comparison of internal target volumes. Values are shown in mean (range)

	ITV_conv_	ITV_GM_	ITV_SRA_				ITV_MRA_
			Lung	Diaphragm	Abdominal wall	Chest wall	
Volume (mL)	14.1 (3.4–47.6)	11.2 (2.2–35.8)	9.6 (2.0–33.3)	9.6 (2.0–33.3)	9.9 (2.0–33.3)	10.5 (2.0–33.3)	8.7 (1.6–33.3)
Difference (%)	–	21.3	31.9	31.9	29.8	25.5	38.3

ITV_SRA_, the internal margins derived from root‐mean‐square error (RMSE) of single regression analysis (SRA) for the lung volume, the displacement of diaphragm, abdominal wall, and chest wall were added to the gross tumor volume (GTV) delineated on 50% phase of four‐dimensional computed tomography (4D‐CT) images; ITV_MRA_, the internal margins derived from RMSE of multiple regression analysis (MRA) were added to the GTV delineated on 50% phase of 4D‐CT images; ITV_GM_, the internal margins of 0.6 mm, 0.9 mm and 0.2 mm in left‐right (LR), anterior‐posterior (AP) and superior‐inferior (SI) directions, respectively, were added to the GTV delineated on 50% phase of 4D‐CT images; ITV_conv_, target volume derived from contouring GTVs on all 10 phases of the 4D‐CT.

## 4 DISCUSSION

Among the anatomical features, such as the lung volume, diaphragm, abdominal wall, and chest wall, the lung volume showed the highest correlation with lung tumor position, followed by displacements of the diaphragm, abdominal wall, and chest wall. This study demonstrated that internal anatomical signals had a higher correlation with lung tumor positions than did external anatomical features. Surrogate signals acquired from multiple anatomical features, as well as the feasibility of using multiple anatomical signals for 4D‐CT or 4D‐RT, were evaluated.

A comparison between the internal and external anatomical signals has been reported by others. Huguet et al. reported that the correlation of a biliary stent, fiducial seed, and RPM marker displacements with pancreatic tumor position using 4D‐CT and an external surrogate signal^11^ They concluded that the biliary stent and fiducial seed were better predictors of tumor motion than was the RPM marker. Additionally, the fiducial stent was slightly better than the biliary stent in predicting tumor motion in the SI direction and clearly better for predicting AP motion. Thus, internal anatomical features have the potential to improve the estimating accuracy of the lung tumor position.

Cervino et al. reported the correlation between lung tumor position and the diaphragm position using fluoroscopic images^12^ They founded that the error between lung tumor position and diaphragm position was 2.1 mm in 95% confidence level. In the current study, it was found that the MRA approach improved estimating accuracy compared with the SRA approach (Fig. 2). Using the MRA approach, the RMSE of the lung tumor position was within 1.6 mm, which enables reducing the internal margin size when applying respiratory motion management techniques (see Fig.2(a)). However, note that overestimating or underestimating tumor motion is possible using 4D‐CT.^5^, ^13^ In an absolute manner, the RMSEs in the SI and 3D directions were larger than those in the LR and AP directions because the average displacements of lung tumor positions were larger in the SI and 3D directions than in the LR and AP directions. In the relative evaluation, the RMSEs in the SI and 3D directions were smaller than those in the LR and AP directions. This implies that the estimated lung tumor position had a high correlation with the actual position in the SI and 3D directions. In the explanatory variables of the MRA approach, the lung volume had a high standard partial regression coefficient (see Fig. 3). The results in Table 2 show that the explanatory variable of the lung volume had a significant difference in the 3D direction compared with the other explanatory variables, showing that the lung volume had a significant influence on the estimated lung tumor position. In current clinical practice, a single anatomical signal and a correspondence model between a single anatomical signal and a motion of the internal anatomy are typically used to reconstruct 4D‐CT^14^ and to estimate target position,^15^, ^16^, ^17^ respectively. In addition, correspondence models have been used to improve image quality in 4D‐CT image reconstruction.^18^, ^19^ From these findings and our results, if the MRA approach is available, using multiple tools including a spirometer or infrared camera, the correspondence model will be improved, which would provide higher accuracy for 4D‐RT and higher image quality for 4D‐CT.

Matsuo et al. conducted RTTT using a gimbal mounted linac for lung cancer and reported that PTV size was reduced by 30.1% compared with conventional PTV.^20^ The intrafractional variation between the centroid of tumor and the centroid of fiducial markers was included in their PTV.^10^ We also found that MRA approach had a potential to reduce internal margin in RTTT without fiducial markers. Currently, markerless tracking technique has been reported by other facilities. Rottmann et al. reported direct RTTT approach that used images on an electronic portal imaging devices to track for lung tumor directly and concluded that the RMSE between tumor and center of field aperture position was within 1 mm.^21^ As the other strategy of markerless tracking, MRA approach would be useful to reduce internal margin for RTTT.

Recently, magnetic resonance (MR)‐guided radiotherapy systems, such as the MRIdian system (ViewRay, Inc., Oakwood Village, OH, USA) and the MR‐linac system using a 6‐MV linear accelerator (Elekta, Crawley, UK) and a 1.5‐T MRI (Philips, Best, The Netherlands), have been of interest^22^, ^23^ Using such a system, anatomical features are observed in real time without additional imaging doses. Feng et al. observed anatomy changes using MR images in real time.^24^ Several anatomical features can be observed simultaneously; thus, the MRA approach can be applied in the estimation of tumor position. Although some lung tumors can be identified directly on MR images, it is difficult to identify those with low density. In general, ground glass opacity lesions often fail to be detected on MR images.^25^ Applying the MRA approach to the MR‐guided system, highly accurate predictions of the position of a lung tumor (even those with low density) should be possible from anatomical features.

## 5 CONCLUSIONS

Lung tumor position was estimated from anatomical features using SRA and MRA approaches and the impact of these approaches on ITVs were assessed. We confirmed that the variance in lung volume had an influence on the estimated lung tumor position. Moreover, multiple anatomical features improved the estimation accuracy of lung tumor position and reduced the ITV by using MRA and SRA approaches, compared with the ITV_conv_.

## ACKNOWLEDGMENTS

We acknowledge financial support by a Grant‐in‐Aid for Scientific Research from the Ministry of Education, Culture, Sports, Science, and Technology of Japan (Grant 25253078).

## CONFLICT OF INTEREST

The authors declare no conflict of interest.
